# High-throughput root phenotyping of crop cultivars tolerant to low N in waterlogged soils

**DOI:** 10.3389/fpls.2023.1271539

**Published:** 2023-09-13

**Authors:** Liping Huang, Yujing Zhang, Jieru Guo, Qianlan Peng, Zhaoyang Zhou, Xiaosong Duan, Mohsin Tanveer, Yongjun Guo

**Affiliations:** ^1^ International Research Center for Environmental Membrane Biology, College of Food Science and Engineering, Foshan University, Foshan, China; ^2^ Foshan ZhiBao Ecological Technology Co. Ltd., Foshan, China; ^3^ Tasmanian Institute of Agriculture, University of Tasmania, Hobart, TAS, Australia

**Keywords:** root phenotyping, root traits, NUE, imaging sensors, waterlogging

## Introduction

**Figure 1:**
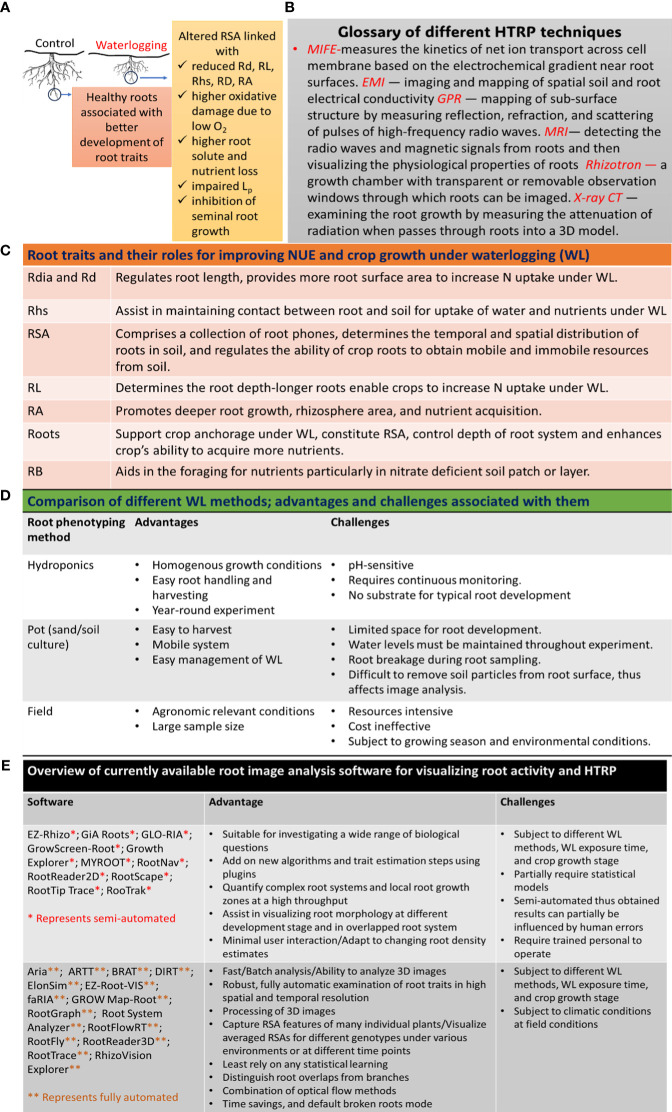
Application of HTRP for studying root systems and improving NUE under WL: **(A)** effects of WL on root growth, **(B)** glossary of different HTRP techniques used for root phenotyping, **(C)** role of different root traits for improving NUE under WL, **(D)** comparison of different WL methods, and **(E)** application of different image analysis software to quantify and visualize root system. Rd, root development; Rdia, root diameter; RL, root length; Rhs, root hairs; RA, root activity; Root*, fine and coarse roots; Lp, root hydraulic conductivity; RB, root branching; MIFE, microelectrode ion flux estimation; EMI, electromagnetic induction; GPR, ground-penetrating radar; MRI, magnetic resonance imaging; X-ray-CT, X-ray computed tomography.

Waterlogging (WL) is one of the most damaging abiotic stresses, affecting 1,700 million hectares of land surface annually (Kaur et al., 2020). Under WL, saturation of soil pores with excessive water results in the development of anaerobic conditions with a subsequent reduction in root growth (**Figure 1A**; Pais et al., 2022). WL induces nutrient imbalances in soil by inducing chemical reduction of some nutrients, including nitrogen (N) (Steffens et al., 2005), thus leading to both nutrient deficiency and/or toxic buildups in soil. N is a very important mineral nutrient and plays a critical role in plant physiology; thus, nitrogen fertilization is adopted as one of the most essential principles for efficient crop production systems (Shah et al., 2021). Nitrogen application boosts crop yield (Shah et al., 2017; Shah et al., 2022); however, excessive application of N comes with several environmental issues. WL promotes soil N losses via runoff, leaching, and denitrification with a concomitant reduction in crop productivity, thus imposing economic and environmental implications. Thus, it is important to understand and improve nitrogen use efficiency (NUE) in plants under WL.

Roots uptake N from the soil in various forms, including amino acids, nitrate (NO_3_^−^), and ammonium (NH_4_^+^); however, NO_3_^−^ is a major source of N in plants (Arduini et al., 2019). WL reduces root N uptake by altering root development (RD), root system architecture (RSA), and N availability in soil. The adoption of advanced agronomic N management techniques, including slow-release fertilizer, biochar application, or inoculation, plays a significant role in improving NUE under WL; however, the efficacy of any agronomic technique greatly depends on soil type and plant species. Moreover, recent development in genetics and breeding techniques have also shown tremendous potential in the development of crop cultivars with higher NUE under low N availability; however, the development of such cultivars is very complex due to genotype and environmental interactions. Moreover, a bottleneck has arisen in the collection of quality phenotypic data to advance crop breeding programs compared with genetic analysis. In this context, adoption of high-throughput root-phenotyping (HTRP) can provide blueprints for breeders to enhance N acquisition in roots under WL.

Several HTRP techniques enable us to phenotype and visualize the root performance under different growth conditions (**Figure 1B**); however, contemporary aboveground canopy-based crop phenotyping (GCCP) techniques account for N-deficiency-induced changes in vegetation index (VI) by measuring photosynthesis, chlorophyll contents, leaf temperature, and stay greenness. However, such GCCP data can be easily camouflaged by the multiple environmental factors that can directly or indirectly influence VI traits. Contrarily, roots being the first line of contact with N and WL, focusing on the establishment of HTRP at least at the early growth stage would be beneficial in determining the genetic basis of NUE in plants under WL.

## Correlation between root traits and NUE

Root system (RS) is very important in the context of N acquisition from soil, and several root traits such as root size, root length (RL), root density (Rd), and root distribution determine N acquisition from soil (**Figure 1C**; Garnett et al., 2009). Crop cultivars with larger RL and Rd uptake more N from soil (Ju et al., 2015), thus reducing N losses under WL. RSA is closely related to N uptake, and crop plants with steeper roots uptake more N from the soil (Zhan & Lynch, 2015). The duration of WL also influences the RS and N uptake (Malik et al., 2001); e.g., short-term WL reduced N uptake only in the bottom layer of the soil-filled pot, while long-term WL resulted in reduced N uptake in both the bottom and top layers of the pot (Dresbøll and Thorup-Kristensen, 2012). Root N uptake was more quickly recovered after short exposure to WL than after long exposure to WL, probably due to the production of new roots (Dresbøll and Thorup-Kristensen, 2012). However, even though N uptake was resumed after recovery from WL, oat roots exhibited reduced root biomass under WL, most likely due to the separation of dead root fragments (Brisson et al., 2002), advanced growth stage during recovery (Arduini et al., 2019), continuous N leakage from root tissues, or other detrimental effects of WL on RS (De San Celedonio et al., 2017). Nonetheless, a cultivar-specific relationship between RS and NUE was observed among two Chinese and one American variety of maize (Ju et al., 2015). The insufficient N uptake by roots under WL could also be due to low availability of N in soil (Nguyen et al., 2018), higher N losses, reduced RD (Brisson et al., 2002), and impaired NO_3_^−^ uptake by roots (Pang et al., 2007). Thus, it is not practically easy to ascertain whether lower N availability to roots is the primary cause of reduced root growth under WL or *vice versa*. Therefore, it is important to consider factors such as cultivars, WL duration, WL method, and plant growth stage when performing HTRP.

## Application of HTRP under waterlogging

Labeling the variations among genotypes and species that uphold improved root traits and integrating them into breeding programs for the development of N-efficient cultivars is a very demanding method. However, studying RSA is very challenging due to the complexity of accurately and precisely phenotyping RS under WL. Several HTRP techniques are being used to understand the relationship between RS and NUE under WL (**Figure 1B**); however, under field conditions, root phenotyping is still handled using a medium- to low-throughput platform (Araus et al., 2022). Different WL methods can also influence root phenotyping (**Figure 1D**). Under controlled conditions, growing plants in hydroponics or gel-based media provides an easy approach to monitoring root morphology; however, this is applicable only in early growth conditions (Langan et al., 2022). Sand culture is another HTRP technique to study RS for improved NUE and performance of root traits using scanners (Paez-Garcia et al., 2015). The pH level of soil and soilless cultures needs to be well monitored, as systems with pH instability and low buffer capacity affect N uptake and RD (Lager et al., 2010). Noninvasive measurements of RS for improved NUE under WL can also be examined using image technology, enabling 2D root growth accompanied by real-time gene expression relating to NUE in roots (Rellán-Álvarez et al., 2015). Other noninvasive techniques, including magnetic resonance imaging (MRI) and X-ray computed tomography (CT) (see glossary in **Figure 1B**), assist in visualizing the physiological properties of roots (Mairhofer et al., 2013; Metzner et al., 2015). Nonetheless, technical complexities and high operation costs make these techniques less useful for large-scale phenotyping. The noninvasive microelectrode ion flux measurements (MIFE) technique was used to perform cell-based phenotyping for revealing QTL associated with hypoxia tolerance in barley (Gill et al., 2017) and understanding the N uptake by measuring the kinetics of NO_3_^−^ and NH_4_^+^ fluxes (Garnett et al., 2003).

At field conditions, several techniques have been applied for performing root phenotyping, such as shovelomics and soil coring (SC). Shovelomics also known as root crown phenotyping, consists of the manual digging and excavation of roots and up to 30 cm of rhizosphere (Wasson et al., 2020). SC also works as shovel omics does to some extent; however, SC consists of the extraction of cores from deeper soil using a corer, with a betting examination of RS (Wasson et al., 2014). For a better view of RS, SC is supplemented with a portable fluorescence imaging system known as BlueBox, which provides automatic root counting using image analysis software (Wasson et al., 2016). Geophysical platforms such as electrical resistance tomography and electromagnetic inductance are used to infer root growth under changes in soil water (Srayeddin & Doussan, 2009; Whalley et al., 2017). Moreover, ground-penetrating radar performs mapping of subsurface soil using radio wave pulses and detects RS under field conditions (Liu et al., 2017; Atkinson et al., 2019).

These HTRP techniques can be ineffective, laborious, and subject to soil conditions (soil types, WL duration, N in soil). Moreover, root extraction under WL is also very difficult due to the breakage of root fragments during extraction; thus, alternate approaches supplement HTRP, including phenotyping of aboveground traits. However, measuring aboveground traits can only infer root growth indirectly (Reynolds et al., 2012; Tracy et al., 2020). To understand root response, examination of the stable isotope composition of N in roots under WL can improve our understanding of the physiological basis of roots and NUE under WL. Having said that, isotopic signatures of oxygen in stem water were used as an indicator of water status in water-stressed roots (Kale Celik et al., 2018). Thus, this approach should be used along with other HTRP techniques.

## Can image-based HTRP be used to phenotype under WL?

Performing HTRP using imaging sensors (IS) and platforms goes on to grow exponentially, easing the bottleneck of root phenotypic data collection (Roitsch et al., 2019). IS such as red, green, and blue (RGB) sensors that take images within the wavelength range of 400–700 nm are termed visible IS, while IS that go beyond the visible wavelength are known as spectral IS (SIS) (Beisel et al., 2018; Bruning et al., 2020). In controlled conditions such as glasshouses or growth chambers, IS range from low-cost cameras to costly custom-made imaging setups (Tovar et al., 2018). Recently, Xia et al. (2019) used hyperspectral and RGB to phenotype WL in rape plants and found promising results. Nonetheless, the use of low-cost cameras may result in image noise; thus, to reduce image noise, image fragmentation must performed (Agata et al., 2007). Imaging plants under WL face other challenges due to the presence of extra water in a pot, which reflects the lights of IS and is due to unwanted algal growth. On the other hand, in field conditions, the use of unmanned ariel vehicles (UAV) and satellite-based imaging are the most popular imaging techniques (Li et al., 2014; Langan et al., 2022). Nonetheless, these imaging techniques also face challenges associated with soil heterogeneity and water drainage, so the use of machine learning (ML) has been suggested along with these imaging techniques to study WL in plants (Zhou et al., 2021). For 2D root images, tip locations have been identified using a deep network-based classifier scanned over an image to produce a location map (Pound et al., 2017). For 3D images, deep learning has been applied to the root–soil segmentation problem, where deep-learned features are used to drive a support vector machine classifying root/soil pixels (Douarre et al., 2016).

As mentioned before, RS plays a very important role in N uptake under WL, and using growth pouches to study root performance under WL or performing root phenotyping using the classical 2D imaging technique (Nagel et al., 2012) does not provide a clear understanding of the root development under WL. Thus, the use of tomographic techniques including CT scanning, MRI, or positron emission tomography has been successfully reported in the study of root phenotyping (Atkinson et al., 2019; Wasson et al., 2020). For instance, X-ray CT scanning was used to visualize the formation of aerenchyma under WL in the roots of barley (Kehoe et al., 2022). Therefore, the application of tomographic techniques can assist in root phenotyping under WL, thereby opening new opportunities for future studies. Though several other methods have been designed for root phenotyping by studying different root traits, including root surface area, crown roots, root length, and root density in soil core (Koyama et al., 2021), there is not any standard root phenotyping method to study different aspects of RSA under WL; therefore, the field of IS exhibits much to extend to the research community. Having said that, several image analysis software are available to quantify and visualize root systems (**Figure 1E**). A new initiative has been established to attempt to harness crop-management synergies using phenotyping, robotics, and computational technologies (http://www.phenorob.de/).

## Conclusion

N fertilization has become the necessity of almost every intensive cropping system, and under WL conditions, crops face N deficiency. Thus, it is imperative to improve the ability of crops to improve NUE under limited N availability. Roots play a critical role in acquiring N from soil; thus, it is important to phenotype RS to highlight the root traits and their relationship with NUE under WL. Given that, the application of HTRP is intensifying due to the technical development and measurement of RS. The utilization of IS and noninvasive measurements of RS can facilitate improving NUE in roots under WL. Advances in ML further benefit analyzing root phenotyping data; however, under field conditions, high‐throughput analysis of root phenotyping remains subtle.

## Author contributions

LH: Conceptualization, Writing – review & editing. YZ: Writing – original draft. JG: Writing – original draft. QP: Writing – original draft. ZZ: Writing – original draft. XD: Writing – review & editing. MT: Conceptualization, Writing – review & editing. YG: Conceptualization, Methodology, Writing – review & editing.

## References

[B1] Agata H. Yamashita A. Kaneko T. (2007). Chroma key using a checker pattern background. IEICE Trans. Inf. Syst. 90 (1), 242–249. doi: 10.1093/ietisy/e90-1.1.242

[B2] Araus J. L. Kefauver S. C. Vergara-Díaz O. Gracia-Romero A. Rezzouk F. Z. Segarra J.. (2022). Crop phenotyping in a context of global change: What to measure and how to do it. J. Integr. Plant Biol. 64 (2), 592–618. doi: 10.1111/jipb.13191 34807514

[B3] Arduini I. Baldanzi M. Pampana S. (2019). Reduced growth and nitrogen uptake during waterlogging at tillering permanently affect yield components in late sown oats. Front. Plant Sci. 10, 1087. doi: 10.3389/fpls.2019.01087 31572410 PMC6751512

[B4] Atkinson J. A. Pound M. P. Bennett M. J. Wells D. M. (2019). Uncovering the hidden half of plants using new advances in root phenotyping. Current. Opin. Biotechnol. 55, 1–8. doi: 10.1016/j.copbio.2018.06.002 PMC6378649 30031961

[B5] Beisel N. S. Callaham J. B. Sng N. J. Taylor D. J. Paul A.-L. Ferl R. J. (2018). Utilization of single-image norMalized difference vegetation index (SI-NDVI) for early plant stress detection. App. Plant Sci. 6, e01186. doi: 10.1002/aps3.1186 PMC6201722 30386712

[B6] Brisson N. Rebiere B. Zimmer D. Renault P. (2002). Response of the root system of a winter wheat crop to waterlogging. Plant Soil 243, 43–55. doi: 10.1023/A:1019947903041

[B7] Bruning B. Berger B. Lewis M. Liu H. Garnett T. (2020). Approaches, applications, and future directions for hyperspectral vegetation studies: An emphasis on yield-limiting factors in wheat. Plant Phenome J. 3, e20007. doi: 10.1002/ppj2.20007

[B8] De San Celedonio R. P. Abeledo L. G. Mantese A. I. Miralles D. J. (2017). Differential root and shoot biomass recovery in wheat and barley with transient waterlogging during pre-flowering. Plant Soil 417, 481–498. doi: 10.1007/s11104-017-3274-1

[B48] Douarre C. Schielein R. Frindel C. Gerth S. Rousseau D. (2016). Deep learning based root-soil segmentation from X-ray tomography images. BioRxiv, 071662. doi: 10.1101/071662

[B9] Dresbøll D. B. Thorup-Kristensen K. (2012). Spatial variation in root system activity of tomato (*Solanum lycopersicum* L.) in response to short and long-term waterlogging as determined by 15 N uptake. Plant Soil. 357, 161–172. doi: 10.1007/s11104-012-1135-5

[B11] Garnett T. Conn V. Kaiser B. N. (2009). Root based approaches to improving nitrogen use efficiency in plants. Plant Cell Environ. 32, 1272–1283. doi: 10.1111/j.1365-3040.2009.02011.x 19558408

[B10] Garnett T. P. Shabala S. N. Smethurst P. J. Newman I. A. (2003). Kinetics of ammonium and nitrate uptake by eucalypt roots and associated proton fluxes measured using ion selective microelectrodes. Func. Plant Biol. 30, 1165–1176. doi: 10.1071/FP03087 32689098

[B12] Gill M. B. Zeng F. Shabala L. Zhang G. Fan Y. Shabala S.. (2017). Cell-based phenotyping reveals QTL for membrane potential maintenance associated with hypoxia and salinity stress tolerance in barley. Front. Plant Sci. 8, 1941. doi: 10.3389/fpls.2017.01941 29201033 PMC5696338

[B13] Ju C. Buresh R. J. Wang Z. Zhang H. Liu L. Yang J.. (2015). Root and shoot traitsforrice varietieswith higher grain yield and higher nitrogen use efficiency at lower nitrogen rates application. Field Crops Res. 175, 47–55. doi: 10.1016/j.fcr.2015.02.007

[B14] Kale Çelik S. Madenoğlu S. Sönmez B. Avağ K. Türker U. Çayci G.. (2018). Oxygen isotope discrimination of wheat and its relationship with yield and stomatal conductance under irrigated conditions. Turk. J. Agric. For. 42, 22–28. doi: 10.3906/tar-1709-31

[B15] Kaur G. Singh G. Motavalli P. P. Nelson K. A. Orlowski J. M. Golden B. R. (2020). Impacts and management strategies for crop production in waterlogged or flooded soils: A review. Agron. J. 112, 1475–1501. doi: 10.1002/agj2.20093

[B16] Kehoe S. Byrne T. Spink J. Barth S. Ng CYK. Tracy S. (2022). A novel 3D X-ray computed tomography (CT) method for spatio-temporal evaluation of waterlogging-induced aerenchyma formation in barley. Plant Phenome J. 5 (1), e20035. doi: 10.1002/ppj2.20035

[B17] Koyama T. Murakami S. Karasawa T. Ejiri M. Shiono K. (2021). Complete root specimen of plants grown in soil-filled root box: sampling, measuring, and staining method. Plant Methods 17, 1-13. doi: 10.1186/s13007-021-00798-3 PMC8454053 34544441

[B18] Lager I. Andréasson O. Dunbar T. L. Andreasson E. Escobar M. A. Rasmusson A. G. (2010). Changes in external pH rapidly alter plant gene expression and modulate auxin and elicitor responses. Plant Cell Environ. 33, 1513–1528. doi: 10.1111/j.1365-3040.2010.02161.x 20444216 PMC2920358

[B19] Langan P. Bernád V. Walsh J. Henchy J. Khodaeiaminjan M. Mangina E.. (2022). Phenotyping for waterlogging tolerance in crops: current trends and future prospects. J. Exp. Bot. 73, 5149–5169. doi: 10.1093/jxb/erac243 35642593 PMC9440438

[B20] Li L. Zhang Q. Huang D. (2014). A review of imaging techniques for plant phenotyping. Sensors 14, 20078–20111. doi: 10.3390/s141120078 25347588 PMC4279472

[B21] Liu X. Dong X. Xue Q. Leskovar D. I. Jifon J. Butnor J. R.. (2017). Ground penetrating radar (GPR) detects fine roots of agricultural crops in the field. Plant Soil 423, 517–531. doi: 10.1007/s11104-017-3531-3

[B22] Mairhofer S. Zappala S. Tracy S. Sturrock C. Bennett M. J. Mooney S. J.. (2013). Recovering complete plant root system architectures from soil via X-ray m-computed tomography. Plant Methods 9, 8. doi: 10.1186/1746-4811-9-8 23514198 PMC3615952

[B23] Malik A. I. Colmer T. D. Lambers H. Schortemeyer M. (2001). Changes in physiological and morphological traits of roots and shoots of wheat in response to different depths of waterlogging. Fun. Plant Biol. 28, 1121–1131. doi: 10.1071/PP01089

[B24] Metzner R. Eggert A. Van Dusschoten D. Pflugfelder D. Gerth S. Schurr U.. (2015). Direct comparison of MRI and X-ray CT technologies for 3D imaging of root systems in soil: potential and challenges for root trait quantification. Plant Methods 11, 17. doi: 10.1186/s13007-015-0060-z 25774207 PMC4359488

[B25] Nagel K. A. Putz A. Gilmer F. Heinz K. Fischbach A. Pfeifer J.. (2012). GROWSCREEN-Rhizo is a novel phenotyping robot enabling simultaneous measurements of root and shoot growth for plants grown in soil-filled rhizotrons. Fun. Plant Biol. 39, 891–904. doi: 10.1071/FP12023 32480839

[B26] Nguyen L. T. Osanai Y. Anderson I. C. Bange M. P. Braunack M. Tissue D. T.. (2018). Impacts of waterlogging on soil nitrification and ammonia-oxidizing communities in farming system. Plant Soil 426, 299–311. doi: 10.1007/s11104-018-3584-y

[B27] Paez-Garcia A. Motes C. Scheible W.-R. Chen R. Blancaflor E. Monteros M. (2015). Root traits and phenotyping strategies for plant improvement. Plants 4, 334–355. doi: 10.3390/plants4020334 27135332 PMC4844329

[B28] Pais I. P. Moreira R. Semedo J. N. Ramalho J. C. Lidon F. C. Coutinho J.. (2022). Wheat crop under waterlogging: potential soil and plant effects. Plants 12, 149. doi: 10.3390/plants12010149 36616278 PMC9823972

[B29] Pang J. Ross J. Zhou M. Mendham N. Shabala S. (2007). Amelioration of detrimental effects of waterlogging by foliar nutrient sprays in barley. Fun. Plant Biol. 34, 221–227. doi: 10.1071/FP06158 32689348

[B47] Pound M. P. Atkinson J. A. Townsend A. J. Wilson M. H. Griffiths M. Jackson A. S.. (2017). Deep machine learning provides state-of-the-art performance in image-based plant phenotyping. Gigascience 6 (10), gix083. doi: 10.1093/gigascience/gix083 PMC5632296 29020747

[B30] Rellán-Álvarez R. Lobet G. Lindner H. Pradier P.-L. Sebastian J. Yee M.-C.. (2015). GLO-Roots: an imaging platform enabling multidimensional characterization of soil-grown root systems. eLife 4, e07597. doi: 10.7554/eLife.07597 26287479 PMC4589753

[B31] Reynolds M. Pask A. Mullan D. (2012). Physiological Breeding, I: interdisciplinary approaches to improve crop adaptation (Mexico: CIMMYT).

[B32] Roitsch T. Cabrera-Bosquet L. Fournier A. Ghamkhar K. JimenezBerni J. Pinto F.. (2019). Review: New sensors and data-driven approaches—A path to next generation phenomics. Plant Sci. 282, 2–10. doi: 10.1016/j.plantsci.2019.01.011 31003608 PMC6483971

[B33] Shah A. N. Iqbal J. Tanveer M. Yang G. Hassan W. Fahad S.. (2017). Nitrogen fertilization and conservation tillage: a review on growth, yield, and greenhouse gas emissions in cotton. Environ. Sci. Poll. Res. 24, 2261–2272. doi: 10.1007/s11356-016-7894-4 27796993

[B34] Shah A. N. Javed T. Singhal R. K. Shabbir R. Wang D. Hussain S.. (2022). Nitrogen use efficiency in cotton: Challenges and opportunities against environmental constraints. Front. Plant Sci. 13, 970339. doi: 10.3389/fpls.2022.970339 36072312 PMC9443504

[B35] Shah A. N. Wu Y. Iqbal J. Tanveer M. Bashir S. Rahman S. U.. (2021). Nitrogen and plant density effects on growth, yield performance of two different cotton cultivars from different origin. J. King Saud Uni-Sci. 33, 101512. doi: 10.1016/j.jksus.2021.101512

[B36] Srayeddin I. Doussan C. (2009). Estimation of the spatial variability of root water uptake of maize and sorghum at the field scale by electrical resistivity tomography. Plant Soil. 319, 185–207. doi: 10.1007/s11104-008-9860-5

[B37] Steffens D. Hutsch B. W. Eschholz T. Losak T. Schubert S. (2005). Water logging may inhibit plant growth primarily by nutrient deficiency rather than nutrient toxicity. Plant Soil Environ. 51, 545. doi: 10.17221/3630-PSE

[B38] Tovar J. C. Hoyer J. S. Lin A. Tielking A. Callen S. T. Castillo E.. (2018). Raspberry Pi-powered imaging for plant phenotyping. App. Plant Sci. 6, e1031. doi: 10.1002/aps3.1031 PMC5895192 29732261

[B39] Tracy S. R. Nagel K. A. Postma J. A. Fassbender H. Wasson A. Watt M. (2020). Crop improvement phenotyping roots: highlights reveal expanding opportunities. Trends Plant Sci. 25, 105–118. doi: 10.1016/j.tplants.2019.10.015 31806535

[B42] Wasson A. Bischof L. Zwart A. Watt M. (2016). A portable fluorescence spectroscopy imaging system for automated root phenotyping in soil cores in the field. J. Exp. Bot. 67, 1033–1043. doi: 10.1093/jxb/erv570 26826219 PMC4753854

[B40] Wasson A. P. Nagel K. A. Tracy S. Watt M. (2020). Beyond digging: non-invasive root and rhizosphere phenotyping. Trends Plant Sci. 25, 119–120. doi: 10.1016/j.tplants.2019.10.011 31791653

[B41] Wasson A. P. Rebetzke G. J. Kirkegaard J. A. Christopher J. Richards R. A. Watt M. (2014). Soil coring at multiple field environments can directly quantify variation in deep root traits to select wheat genotypes for breeding. J. Exp. Bot. 65, 6231–6249. doi: 10.1093/jxb/eru250 24963000 PMC4223987

[B43] Whalley W. R. Binley A. Watts C. W. Shanahan P. Dodd I. C. Ober E. S.. (2017). Methods to estimate changes in soil water for phenotyping root activity in the field. Plant Soil. 415, 407–422. doi: 10.1007/s11104-016-3161-1 32025056 PMC6979655

[B44] Xia J.A. Cao H. Yang Y. Zhang W. Wan Q. Xu L.. (19). Detection of waterlogging stress based on hyperspectral images of oilseed rape leaves (Brassica napus L.). Com. Electron. Agric. 59–68. doi: 10.1016/j.compag.2019.02.022

[B45] Zhan A. Lynch J. P. (2015). Reduced frequency of lateral root branching improves N capture from low-N soils in maize. J. Exp. Bot. 66, 2055–2065. doi: 10.1093/jxb/erv007 25680794 PMC4378636

[B46] Zhou J. Mou H. Zhou J. Ali M. L. Ye H. Chen P.. (2021). Qualification of soybean responses to flooding stress using UAV-based imagery and deep learning. Plant Phenomics 2021, 1–13. doi: 10.34133/2021/9892570 PMC8261669 34286285

